# Intraoperative neurophysiological monitoring for motor function preservation during AVMs resection: Indication or redundancy? Beyond the doctrine of *“all-or-nothing”*

**DOI:** 10.1007/s10143-026-04146-8

**Published:** 2026-01-30

**Authors:** Carmelo Lucio Sturiale, Fulvio Grilli, Gianluca Trevisi, Matteo Palermo, Michele Di Domenico, Marco Galeazzi, Rina di Bonaventura, Alessandro Olivi, Alessio Albanese

**Affiliations:** 1 Department of Neurosurgery, Fondazione Policlinico Universitario Agostino Gemelli IRCCS, Università Cattolica del Sacro Cuore, Rome, Italy; 2 Department of Neurosciences, Imaging and Clinical Sciences, G. D’Annunzio University, Chieti-Pescara, Italy. Neurosurgical Unit, Ospedale Civile Spirito Santo, Pescara, Italy; 3 Institute of Neurosurgery, Fondazione Policlinico Universitario “A. Gemelli”, Università Cattolica del Sacro Cuore , L.go A. Gemelli, 8–00168 Rome, Italy

**Keywords:** Arteriovenous malformation, IONM, Neuromonitoring, SSEP, MEP, Eloquent, Mapping

## Abstract

The resection of cerebral arteriovenous malformations (AVMs) involving eloquent motor regions remains one of the most challenging tasks in neurosurgery due to the risk of irreversible motor deficits. Historically, such lesions have often been managed conservatively or with radiosurgery, particularly when located near primary motor cortex or corticospinal tracts. However, the advent of intraoperative neurophysiological monitoring (IONM) allows for real-time feedback on motor pathway function, potentially enabling safer resections in areas previously considered inoperable. This study investigates the effectiveness of IONM in reducing motor morbidity during AVM surgery. We prospectively enrolled 48 patients who underwent AVM resection between 2018 and 2025 at a single center. All surgeries incorporated a standardized IONM protocol, including continuous somatosensory evoked potentials (SSEPs), motor evoked potentials (MEPs), and dynamic cortical and subcortical mapping. Patients were stratified into high and low motor-risk groups based on AVM topography. Clinical, imaging, and angioarchitectural data were analyzed. Primary outcomes were new postoperative motor deficits; secondary outcomes included modified Rankin Scale (mRS) scores and residual nidus presence. Statistical analysis included univariate and multivariate regression. Of the 48 patients (mean age 45.2), 42% presented with hemorrhage and 50% had left-hemisphere AVMs. Spetzler-Martin grades ranged from I to IV. Three patients (6.25%) had residual AVM postoperatively, two of which were intentional due to IONM alerts. None developed new deficits. One residual spontaneously thrombosed during follow-up. Multivariate analysis showed preoperative mRS, not motor eloquence, was the main predictor of outcome in ruptured AVMs. No significant difference in postoperative motor function or mRS was found between high- and low-risk groups. IONM enables safe, function-preserving AVM resection in motor eloquent areas. It supports intraoperative decision-making, reducing morbidity and expanding surgical indications guiding a “maximal safe resection” strategy focused on functional preservation rather than angiographic radicality alone.

## Introduction

The surgical resection of cerebral arteriovenous malformations (AVMs) remains a considerable challenge in neurosurgery, primarily due to the imperative of achieving complete nidus obliteration while preserving neurological function. AVMs located within or adjacent to eloquent motor areas pose a particularly high risk of postoperative neurological deficits preservation in selected cases [1–3]. Similarly, AVMs associated with large proximal vessels of the middle cerebral artery (MCA) and anterior cerebral artery (ACA), which may function merely as transit or bystander arteries, further complicate surgical management [4–6].

Historically, in fact, AVMs in eloquent areas, have been mainly managed conservatively or with stereotactic radiosurgery to minimize surgical morbidity [7, 8].

On the other hand, in contemporary neurosurgical practice, the presence of a lesion in an eloquent area is no longer seen as a strict contraindication to surgery and this is increasingly true also for AVMs.

Advances such as preoperative MRI tractography and planning along with various adjunctive intraoperative tools have been developed to enhance surgical precision and safety. Among them, the most important has been the development of the ability to perform intraoperative neurophysiological mapping and monitoring (IONM) of cortex and subcortical fibers, which radically have transformed the surgical strategies influencing the intraoperative decision-making, reducing the surgical risks and improving the clinical outcomes [9–11].

Over the past five years, we have systematically implemented the IONM in all AVM resections to evaluate its impact on the extent of resection and the incidence of new postoperative motor deficits.

## Materials and methods

We prospectively enrolled 48 patients who underwent surgical treatment for ruptured or unruptured arteriovenous malformations (AVMs) between 2018 and 2025 at the Fondazione Policlinico Universitario A. Gemelli IRCCS, Rome, Italy.

A standardized IONM protocol was utilized for all procedures. The protocol included:

1. Cortical and Subcortical Mapping: Dynamic mapping was employed to identify and preserve critical motor pathways and functional cortex.
2. Continuous Sensori-Motor Pathway Monitoring: Somatosensory evoked potentials (SSEPs) and motor evoked potentials (MEPs) were monitored continuously throughout the AVM resection to ensure the real-time integrity of sensorimotor neural pathways.

For each patient, demographic, clinical, and angioarchitectural data related to the AVM were systematically collected, including nidus location, arterial supply, venous drainage, and their relationship to eloquent motor structures.

Patients were prospectively stratified into two groups according to motor functional risk using a predefined, anatomy-based framework. High motor-risk AVMs were defined by at least one of the following features: (i) a nidus located within or immediately adjacent to peri-motor regions, including the primary motor cortex, supplementary motor area, or subcortical motor pathways; and/or (ii) arterial supply arising from large proximal branches of the middle cerebral artery (MCA) or anterior cerebral artery (ACA), particularly within the sylvian or interhemispheric fissures, where critical bystander vessels may supply eloquent motor territories. Classification in risk groups was solely based on routine MRI findings.

Low motor-risk AVMs were defined as lesions located at a distance from motor eloquent areas and predominantly supplied by distal or terminal arterial branches, without involvement of major proximal feeders or bystander vessels supplying motor pathways.

This stratification was defined a priori and was independent of Spetzler–Martin grading, aiming to capture procedure-specific motor vulnerability rather than anatomical complexity alone.

At the time of surgery, IONM was routinely used in all AVMs resection as part of a standardized armamentarium in our institutional protocol, independently from motor-risk classification. In general, in high motor-risk AVMs, a full multimodality monitoring setup including SSEPs, MEPs, and cortical/subcortical mapping when indicated was systematically used.

In low motor-risk AVMs, instead, IONM was primarily employed as a safety monitoring tool, most commonly using SSEPs to detect unexpected ischemic events during temporary arterial feeders occlusion or deep dissection, rather than for functional mapping of eloquent cortex.

Follow-up protocols for all patients included MRI and DSA before hospital discharge, CTA at 6-months follow-up and a new DSA at 1-year follow-up.

### Anesthetic protocol and monitoring protocol

All patients received total intravenous anesthesia (TIVA) with Propofol and remifentanil. Inhalation agents and long-acting neuromuscular agents were avoided to ensure reliable MEP and SSEP recordings. Transcortical electrical stimulation was routinely used for MEP monitoring in all cases. If transcranial MEPs were lost or significantly deteriorated during cortical surgery, a subdural strip electrode was placed, or utilized if already present, and MEP monitoring was continued using direct cortical stimulation, allowing a more focal and reliable assessment of motor pathway function. In cases where the lesion was suspected to be in close proximity to subcortical motor pathways, subcortical stimulation was additionally performed to assess corticospinal tract integrity and proximity. An intraoperative alert was defined as a persistent reduction of ≥ 50% in signal amplitude compared with baseline values for both MEPs and SSEPs. For SSEPs, latency changes were also considered, with a latency increase of ≥ 10% regarded as clinically significant.

### Outcome measures and follow-up

The primary outcome was the incidence of new postoperative motor deficits. Motor function was assessed preoperatively, in the immediate postoperative period, at hospital discharge, and during follow-up visits. New-onset postoperative motor deficits were classified as transient if complete recovery occurred within six months, and as permanent if deficits persisted beyond six months of follow-up. Secondary outcomes included postoperative functional status, assessed using the modified Rankin Scale (mRS), and the rate of residual nidus after surgery. Patients with confirmed residual nidus on postoperative imaging were followed longitudinally to monitor for subsequent hemorrhage, nidus growth, or spontaneous thrombosis.

### Statistical analysis

All statistical analyses were performed using JASP software (Version 0.19.3). A p-value of less than 0.05 was considered statistically significant.

Univariate analysis was used to compare baseline characteristics and outcomes between the high-risk and low-risk groups. The Student’s t-test or Welch’s t-test was used for continuous variables, while the Chi-Squared test or Fisher’s exact test was used for categorical variables. A logistic regression model was developed to identify predictors of new postoperative motor deficits.

To assess functional outcomes, a linear regression model was used to evaluate the relationship between motor-risk category and postoperative mRS scores, analyzed separately for subgroups of patients with ruptured and unruptured AVMs.

Finally, a univariate analysis was conducted to investigate the association between the presence of a residual nidus and potential risk factors, including high motor-risk location, hemorrhagic presentation, and the occurrence of intraoperative seizures.

### Ethical statement and consent

Ethics approval was not required for this retrospective review, as all data were collected from routine clinical care and anonymized prior to analysis. No identifiable patient information is presented. As such, consent for publication was not applicable.

## Results

We prospectively enrolled 48 patients with ruptured or unruptured arteriovenous malformations (AVMs) treated between 2018 and 2025. Demographic, clinical and angioarchitectural characteristics of patients and AVMs are shown in Table 1.

**Table 1:** Demographic, clinical and angioarchitectural characteristics of patients and AVMs

Variable	Total patients = 48
Sex	31 Females (64.6%)
Age (years)	45.2 (± 16)
Left hemisphere	Total = 24 (50%)
Left frontal	12 (46.2%)
Left temporal	4 (15.4%)
Left parietal	4 (15.4%)
Left occipital	2 (7.7%)
Left insula	1 (3.8%)
Left temporo-parietal	1 (3.8%)
Right hemisphere	Total = 22 (45.8%)
Right frontal	10 (45.5%)
Right temporal	4 (18.2%)
Right parietal	4 (18.2%)
Right occipital	2 (9.1%)
Right fronto-parietal	1 (4.5%)
Right temporo-insular	1 (4.5%)
Cerebellar	Total = 2 (4.2%)
Spetzler-martin grade	Mean = 1.89 (± 0.86)
Grade 1	18 (37.5%)
Grade 2	19 (39.6%)
Grade 3	9 (18.8%)
Grade 4	2 (4.2%)
Lawton grade	Mean = 3.32 (± 0.86)
Grade 1	1 (2.1%)
Grade 2	6 (12.5%)
Grade 3	20 (41.7%)
Grade 4	18 (37.5%)
Grade 5	3 (6.3%)
Arterious aneurysms	17 (35.4%)
Venous aneurysms	16 (33.3%)
Mean nidus size (mm)	21.3 (± 10.9)
Nidus type - compact	37 (77.1%)
Nidus type - diffuse	11 (22.9%)
Venous drainage - superficial	35 (72.9%)
Venous drainage - deep	13 (27.1%)
Venous stenosis	7 (14.6%)
Direct shunt	15 (31.3%)
Cortical AVM	34 (70.8%)
Subcortical AVM	32 (66.7%)
AVM with motor-related risk	Total = 21 (43.7%)
Motor cortex	1 (2.8%)
Anatomical or Vascular involvement of CST	11(22.9%)
Both	9 (18.8%)
AVM not at motor-related risk	Total = 27 (56.2%)
Calcarine cortex	4 (8.3%)
Language related cortex	5 (10.4%)
Other non-eloquent areas	18 (37.5%)
Ruptured presentation	20 (42%)
Post-operative (micro) residual nidus	3 (6.2%)

According to the predefined anatomy-based motor risk stratification, 21 of 48 AVMs (43.8%) were classified as high motor-risk, while 27 AVMs (56.2%) were classified as low motor-risk.

Among unruptured AVMs, 12/28 lesions (42.9%) were at higher motor risk, whereas 16/28 (57.1%) were at lower motor risk.

Similarly, among ruptured AVMs, 9/20 lesions (45%) were classified as high motor-risk and 11/20 (55%) as low motor-risk.

The cohort included 31 females (64.6%) and 17 males (35.4%), with a mean age of 45.2 years (± 16). Overall, 20/48 AVMs (42%) had a hemorrhagic clinical presentation.

Regarding nidus topography, 24 AVMs (50%) were in the left hemisphere: 12 frontal (46.2%), 4 temporal (15.4%), 4 parietal (15.4%), 2 occipital (7.7%), 1 insular (3.8%), and 1 temporo-parietal (3.8%). Twenty-two AVMs (45.8%) were in the right hemisphere: 10 frontal (45.5%), 4 temporal (18.2%), 4 parietal (18.2%), 2 occipital (9.1%), 1 fronto-parietal (4.5%), and 1 temporo-insular (4.5%). Two AVMs (4.2%) were in the cerebellum.

According to the Spetzler-Martin grading system, 18 AVMs (37.5%) were grade I, 19 (39.6%) grade II, 9 (18.8%) grade III, and 2 (4.2%) grade IV, with a mean score of 1.89 (± 0.86). Based on the Lawton supplementary grading system, 1 AVM (2.1%) was grade I, 6 (12.5%) grade II, 20 (41.7%) grade III, 18 (37.5%) grade IV, and 3 (6.3%) grade V, with a mean score of 3.32 (± 0.86).

Flow-related aneurysms were identified in 17 patients (35.4%), while venous aneurysms were present in 16 (33.3%). The nidus was classified as compact in 37 cases (77.1%) and diffuse in 11 (22.9%), with a mean overall size of 21.3 mm (± 10.9). Superficial venous drainage was observed in 35 cases (72.9%) and deep venous drainage in 13 (27.1%). Venous stenosis was detected in 7 patients (14.6%).

**Fig. 1:**
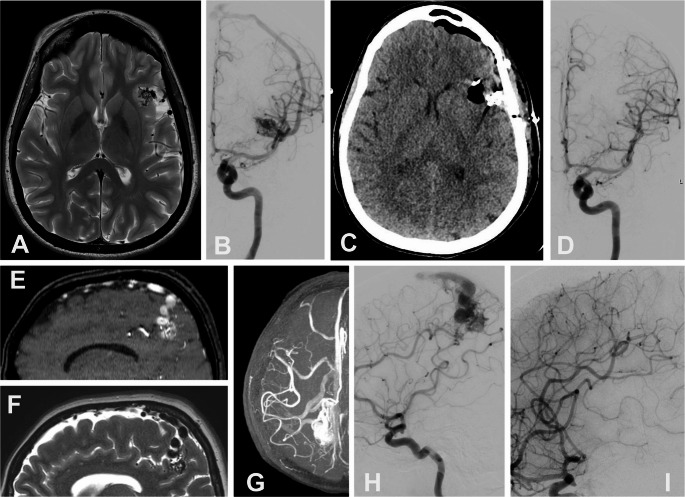
The figure shows the two types of AVM with high motor risk. The first has a nidus located in the left fronto-insular cortex (**A**), supplied by small branches of the middle cerebral artery (**B**) and is connected to proximal bystander branches not involved in the feeding of the malformation. IONM has proven useful during temporary clipping until complete resection of the malformation avoiding the sacrifice of bystanders and transit arteries (**C**, **D**). The second shows a post-Rolandic parietal AVM bordering the central fissure and in direct contact with the primary motor cortex (**E**, **F**, **G**, **H**) that was completely dissected from the eloquent areas with the aid of IONM and removed without motor deficits (**I**)

**Fig. 2:**
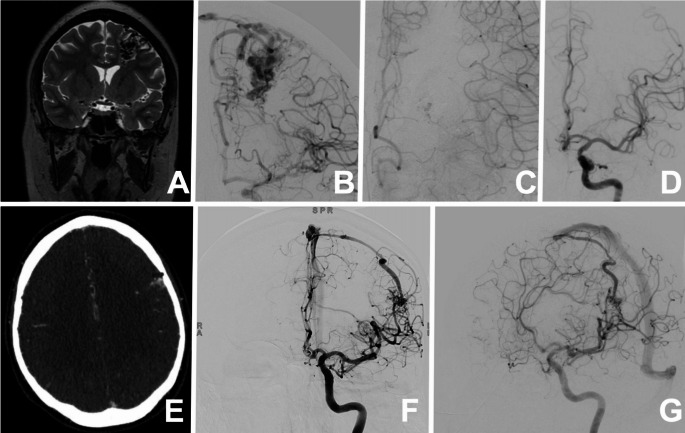
(**A**) Magnetic resonance imaging (MRI) demonstrating an arteriovenous malformation (AVM) located in the perirolandic territory. (**B**) Digital subtraction angiography (DSA) of the same lesion, showing arterial feeders arising from branches of the left middle cerebral artery and anterior cerebral artery. (**C**) Postoperative angiography revealing a minimal deep residual lesion adjacent to the internal capsule. (**D**) Six-month follow-up angiography demonstrating spontaneous thrombosis of the residual nidus. (**E**) Second case: non-contrast CT showing a small AVM in contact with proximal branches of the ipsilateral middle cerebral artery, which provide its arterial supply. (**F**) Corresponding angiographic image of the second case. (**G**) Postoperative angiography showing a minimal residual AVM, intentionally left due to transient loss of neurophysiological potentials during temporary clamping of the afferent vessels; the patient is currently under radiological follow-up with no evidence of new hemorrhages

A small residual nidus was observed postoperatively in 3 patients; in two of these cases, the residual was intentionally left due to intraoperative neurophysiological monitoring (IONM) alerts indicating risk to motor function (Figs. 1 and 2).

The univariate analysis comparing ruptured and unruptured AVMs, showed that the two groups were similar for demographics, AVM side and size; moreover, the difference in terms of nidus topography with higher and lower motor risk was not statistically significant between the two groups and, as a consequence, pre- and post-operative mRS was significantly higher in the ruptured AVM due to the persistence of motor deficit after bleeding that in fact showed a trend (Table 2).

**Table 2:** Univariate analysis comparing different variables between ruptured and unruptured AVMs

Variable	Unruptured AVM (*n* = 28)	Ruptured AVM (*n* = 20)	*p*
Age (years)	43.5 (± 15.8)	47.5 (± 16.6)	0.4
Female sex	20 (71%)	11 (55%)	0.2
Left hemisphere	14 (50%)	12 (60%)	0.5
Spetzler-Martin grade	1.9 (± 0.9)	1.9 (± 0.9)	0.7
Lawton grade	3.6 (± 0.8)	2.9 (± 0.8)	0.002*
Nidus size (mm)	22.7 (± 9.9)	19.4 (± 12.2)	0.3
At Motor Risk	12 (43%)	9 (45%)	0.9
Pre-operative mRS	0.1 (± 0.4)	2.1 (± 1.6)	< 0.001*
Post-operative mRS	0.3 (± 0.5)	1.1 (± 1.1)	0.01*
Pre-operative seizures	10 (36%)	9 (45%)	0.5
Pre-operative motor deficit	1 (3.5%)	5 (25%)	0.07
Intra-operative seizures	2 (7%)	1 (5%)	1
Post-operative seizures	3 (11%)	4 (20%)	0.4
Post-operative new-onset motor deficits	2 (7%)	1 (5%)	1

In patients with unruptured AVMs (12 high motor-risk vs. 16 low motor-risk lesions), univariate analysis showed no significant differences in demographic, clinical, or angioarchitectural characteristics between the two groups. No differences were observed in postoperative clinical outcomes, including mRS score and incidence of new motor deficits (Table 3).

**Table 3:** Univariate analysis comparing different variables between AVMs at higher and lower motor risk in unruptured AVMs

Variable	Motor Risk (*n* = 12)	Non-Motor Risk (*n* = 16)	*p*
Age (years)	48.3 (± 14.5)	39.9 (± 16.2)	0.2
Female sex	8 (67%)	12 (75%)	0.7
Left hemisphere	4 (33.3%)	10 (62.5%)	0.3
Spetzler-Martin grade	2.1 (± 0.8)	1.8 (± 0.9)	0.4
Lawton grade	3.8 (± 0.6)	3.6 (± 0.9)	0.5
Nidus size (mm)	20.3 (± 12.2)	24.4 (± 7.8)	0.3
Pre-operative mRS	0.2 (± 0.4)	0.1 (± 0.3)	0.8
Post-operative mRS	0.3 (0.5)	0.3 (± 0.4)	0.6
Pre-operative seizures	4 (33.3%)	6 (37.5%)	0.8
Pre-operative motor deficit	1 (8.3)	0	0.4
Intra-operative seizures	2 (16.7%)	0	0.2
Post-operative seizures	1 (8.3%)	2 (12.5%)	1
Post-operative new-onset motor deficits	2 (16.7%)	0	0.2

In the subgroup of ruptured AVMs (9 high motor-risk vs. 11 low motor-risk lesions), postoperative mRS was significantly higher in the high motor-risk group. This difference reflected a worse preoperative neurological status, driven by a significantly higher incidence of preoperative motor deficits following hemorrhage, rather than surgery-related morbidity (Table 4).

**Table 4:** Univariate analysis comparing different variables between AVMs at higher and lower motor risk in ruptured AVMs

Variable	Motor Risk (*n* = 9)	Non-Motor Risk (*n* = 11)	*p*
Age (years)	47.8 (± 12.9)	47.3 (± 19.7)	0.9
Female sex	5 (56%)	6 (54.5%)	1
Left hemisphere	7 (78%)	5 (45.5%)	0.2
Spetzler-Martin grade	2.2 (± 0.4)	1.5 (± 1)	0.08
Lawton grade	3 (± 0.7)	2.8 (± 0.9)	0.6
Nidus size (mm)	22 (± 15.7)	17.4 (± 8.6)	0.4
Pre-operative mRS	2.8 (± 1.9)	1.6 (± 1.2)	0.1
Post-operative mRS	1.8 (± 1.2)	0.5 (± 0.7)	0.006*
Pre-operative seizures	4 (44%)	5 (45.5%)	1
Pre-operative motor deficit	5 (56%)	0	0.008*
Intra-operative seizures	1 (11%)	0	0.45
Post-operative seizures	1 (11%)	3 (27%)	0.6
Post-operative new-onset motor deficits	1 (11%)	0	0.45

Multivariate analyses confirmed that high motor-risk AVM location was not independently associated with the occurrence of new postoperative motor deficits nor with worse functional outcome at follow-up (Tables 5 and 6).

**Table 5:** Logistic regression with post-operative new motor deficit as dependent variable

Coefficients					
				95% CI (OR scale)	
Variable	Standard Error	Odds Ratio	p	Lower	Upper
Age	0.064	0.946	0.388	0.834	1.073
Nidus size (mm)	0.062	1.115	0.079	0.988	1.258
Bleeding onset (y)	1.777	0.261	0.450	0.008	8.491
Motor Risk (y)	5047.913	5.262 × 10^+ 8^	0.997	0.000	∞

**Table 6:** Linear regression with post-operative modified Rankin scale as dependent variable (limited to the unruptured AVMs group)

Coefficients				95% CI	
Variable	Unstandardized	Standard Error	p	Lower	Upper
Age	0.005	0.006	0.401	-0.007	0.017
Nidus size (mm)	0.013	0.010	0.195	-0.007	0.033
Side (L)	0.272	0.192	0.170	-0.125	0.670
Motor Risk (y)	0.174	0.200	0.393	-0.239	0.587

In ruptured AVMs, preoperative clinical severity, as measured by the mRS score, emerged as the main determinant of outcome, whereas motor-risk topography did not retain independent prognostic significance (Table 7).

**Table 7:** Linear regression with post-operative modified Rankin scale as dependent variable (limited to the ruptured AVMs group)

Coefficients				95% CI	
Variable	Unstandardized	Standard Error	p	Lower	Upper
Age	0.010	0.011	0.369	-0.013	0.033
Nidus size (mm)	0.013	0.016	0.445	-0.022	0.047
Side (L)	-0.031	0.367	0.934	-0.819	0.756
Motor Risk (y)	0.805	0.379	0.052	-0.007	1.617
mRS pre	0.408	0.126	0.006*	0.138	0.678

At a mean follow-up of one year, none of the patients with residual nidus experienced new hemorrhagic events. One of the three residual niduses showed spontaneous thrombosis at 6-month follow-up at digital subtraction angiography (DSA).

### Intraoperative neurophysiological findings during nidus resection

Intraoperative neurophysiological monitoring was successfully performed in all patients using a standardized protocol. No permanent or severe IONM alterations were recorded during AVM resection.

In one patient with a high motor-risk AVM supplied by proximal branches of the middle cerebral artery, a transient decrease in MEP amplitude was observed following temporary clipping of an arterial feeder. Prompt removal of the temporary clip resulted in rapid recovery of MEPs, and resection was continued without further neurophysiological deterioration.

In another case involving a nidus located in the post-Rolandic region, corresponding to the primary somatosensory cortex, a transient reduction in evoked potentials was observed during nidus dissection. This change did not translate into any postoperative motor deficit.

These findings, together with two additional cases in which cortical mapping demonstrated close adjacency between the nidus margin and the precentral gyrus, guided a conservative subarachnoid dissection strategy rather than a subpial resection strategy.

Postoperative digital subtraction angiography demonstrated a small residual AVM in these cases, appearing as an early arteriovenous shunt with persistent arterialization of a small draining vein. This residual component had not been clearly identified intraoperatively, as further dissection in this region was intentionally limited in response to IONM alerts. During follow-up, one of these residual shunts showed spontaneous thrombosis, and no patient developed new postoperative neurological deficits.

## Discussion

In our series of 48 AVM patients, the implementation of IONM appears to mitigate the risk traditionally associated with the resection of high-risk motor AVMs. In fact, patients with AVMs located in those high-risk topographies, such as the peri-motor cortex, the deep locations close to the internal capsule, the interhemispheric region, or the sylvian fissure adjacent to major branches of the MCA and ACA, did not experience an increased incidence of postoperative motor deficits compared to the less eloquent counterpart [1, 3, 14–17]. Indeed, postoperative functional outcomes were similarly favorable in both groups, indicating that the use of IONM effectively “downgraded” the risk profile of motor eloquent AVMs reducing intraoperative uncertainty and supporting real-time, function-guided decision-making. Importantly, IONM should be viewed as an adjunct that enhances safety, without negating the inherent vulnerability of peri-Rolandic regions, particularly in the setting of intraoperative rupture or abrupt hemodynamic changes.

Notably, several AVMs classified as Spetzler–Martin grade I were traditionally considered at lower risk based on angioarchitectural feature. Instead, our results suggest that lesion size or the Spetzler–Martin grading alone is not a sufficient criterion to rule out the need for IONM. Instead, the indication for monitoring should be individualized and based on lesion topography and vascular relationships, including the presence of critical bystander vessels supplying eloquent or functionally relevant brain regions. The principal advantage of IONM stands in the real-time feedback in adjusting the surgical strategy when dissecting the nidus from eloquent cortices or crucial white-matter tracts [18–25]. Similarly, IONM aids surgeons to time the temporary clipping of presumed feeding arteries when distinguishing among bystanders, transit or terminal vessels.

Although, IONM adoption is a widely accepted practice in tumor surgeries and aneurysm clipping, the implementation in AVMs resection has never took hold among vascular neurosurgeon, due to the affirmation of a sort of *“all-or-nothing”* idea for this kind of surgery, which essentially comes down to either accept the significant risk of deficit by pursuing complete resection, or avoid surgery altogether, opting instead for conservative management or radiosurgery [20–23, 26, 27].

One of the main reasons that led to the rooting of this doctrine was that the incomplete resection was considered a failure, and the risk of hemorrhage from residual nidus is retained inacceptable [1, 5–9, 24, 28, 29]. Thus, neuromonitoring feedback was thought to not influencing the surgical objective.

In the last five years, based on the experience gained in oncological surgery and aneurysm clipping, we have abandoned that old attitude and have started to systematically use IONM also in AVM surgery [1, 5–9, 24, 28, 29]. The result has been a paradigm shift in our primary goal, which nowadays is the maximal safe resection with complete functional preservation, rather than total resection at any cost. Moreover, this was almost always possible in our series, especially in cases of Rolandic AVMs, as in such instances the motor cortical areas were slightly dislocated in nearby cortical territories by the presence of the dysplastic nidus [30–35]. In fact, in a minimal percentage of cases, the IONM has pushed us to pursue an in situ deafferentation to safeguard the explored function – an approach increasingly endorsed also by other experienced neurosurgeons in the worldwide. IONM in fact provides the surgeon with continuous functional feedback, allowing dynamic decision-making about how far to proceed [16, 17, 25, 29, 30, 36, 37].

Gabarrós et al. (2011), for example, used cortical mapping in 12 eloquent AVMs achieving a safe complete resection in 67% of cases; in the remaining one-third of patients, the nidus was intentionally left partially in situ and later obliterated with radiosurgery. They concluded that mapping can identify functional cortex and impact the decision to resect the AVM completely, advocating subtotal microsurgical removal followed by radiosurgery as a viable alternative in cases where full resection would pose unacceptable functional risks [7].

Our practice nowadays mirrors this philosophy: in 3 of our cases, IONM warnings prompted us to halt dissection and leave a tiny residual when we considered an excessively high motor risk in continuing with the resection.

All three patients hesitated neurologically intact after surgery and none of the small residuals hemorrhaged during follow-up (mean 1 year), and one residual even spontaneously thrombosed on 6-month follow-up DSA. As regards the other two cases, one with a small Rolandic residual nidus was planned to undergo radiosurgery, while the other is still under conservative follow-up.

Although the short- to mid-term follow-up of patients with small residual nidi was uneventful, the relatively limited observation period represents an important limitation of this study. The long-term natural history and hemorrhagic risk of residual AVM tissue, even when minimal, remain incompletely defined. Therefore, the absence of adverse events in our cohort should be interpreted with caution, and longer longitudinal follow-up is required to definitively confirm the safety of leaving residual nidi in situ. Future studies with extended follow-up and larger cohorts will be essential to clarify this issue.

Of course, this two-staged approach must be weighed against the theoretical risk of hemorrhage from the residual nidus in the interim; however, the same risk profile is commonly accepted whenever radiation treatment is proposed or, although nowadays obsolete, endovascular obliteration stand-alone via the arterial route, since angiographic non-filling cannot be considered with certainty as a biological cure for the disease [16, 17, 25, 29, 30, 36, 37].

Indeed, if same approach is progressively adopted by a growing number of authors who also report follow-up data on the residual nidus, it will be possible to verify whether these small residual niduses really remain hemodynamically active maintaining a high hemorrhagic risk, or the important flow reduction will lead them to spontaneous obliteration, thus validating its safety and efficacy profile.

This finding held true in case of ruptured AVMs, which despite having a worse baseline clinical status that was an independent predictor for final outcome at the multivariate analysis, showed that only the preoperative clinical status, but not the motor eloquent topography, influenced the final outcome [30, 33].

### Mapping and monitoring techniques

Intraoperative mapping and monitoring provide several benefits that together enhance the safety of AVM microsurgery. Cortical mapping with monopolar or bipolar electrode can be used at the start of the resection to localize motor cortex when adjacent to the nidus. This is crucial in cases where chronic ischemia due the steal phenomenon from the AVM has caused functional reorganization or displacement of eloquent cortex [12, 15]. Mapping helps delineating safe entry corridors and margins of resection, making sure of not damaging inadvertently critical cortical areas. IONM pushes to refine the surgical technique from a nidus resection to a nidus dissection.

In our practice, pre-resection mapping often confirmed the expected anatomy from preoperative DTI, but in some cases revealed a displacement or reorganization of motor function secondary to chronic steal phenomenon [5, 10, 11, 14, 17, 28]. As the resection progresses deeper, subcortical stimulation mapping and continuous MEP/SSEP monitoring guide the trajectory of dissection. In agreement, Chang et al. (1999) reported that in 54 AVM surgeries with SSEP monitoring, only 9% showed significant intraoperative SSEP changes; prompt intervention in those cases prevented permanent injury, and 91% of patients without SSEP changes remained neurologically intact. Notably, SSEP alterations in that study predicted postoperative neurological deficits with 86% sensitivity and 98% specificity [13, 33, 38]. More recently, Zhou et al. (2017) compared AVM resections with vs. without IONM and found no overall difference in neurologic outcome between the groups, but a significant reduction in postoperative hemiplegia for Spetzler-Martin grade III AVMs treated with IONM. In Zhou’s monitored cohort, IONM achieved 86.7% sensitivity and 100% specificity for predicting short-term neurologic deficits. Notably, stable SSEPs were 100% specific for intact motor function, although SSEP monitoring alone had lower sensitivity (82%). This shows that stable SSEP signals are reassuring but do not entirely exclude the risk of neurological injury [37]. Therefore, the incorporation of MEP monitoring is essential, as MEPs assess corticospinal tract integrity in real time and offer greater sensitivity to impending motor pathway ischemia compared to SSEPs. For instance, Šmigoc et al. (2023) noted that combining MEP and SSEP monitoring offers superior predictive accuracy for motor deficits than either modality alone. In their series, MEP alerts had no false-positives in indicating corticospinal compromise, although a couple of patients with postoperative weakness had no MEP change, illustrating rare false-negatives [36].

A common intraoperative dilemma arises when encountering an artery that appears to enter the nidus. In this case distinguishing between a direct feeder and an indirect feeder remains a challenge. Indirect feeders often give rise to en passage branches, also known as ‘transit’ or bystander vessels, that after supplying the AVM nidus, continue distally to perfuse normal brain tissue [5, 9, 13]. Temporarily, occluding such vessels to determine whether they perfuse eloquent brain or solely the AVM carries significant risk and calls for caution. Here, neuromonitoring is invaluable: during temporary occlusion of a suspected feeder, we closely watch MEPs and SSEPs for any changes. IONM can confirm if a vessel is truly expendable (direct AVM feeder) or if it also perfuses eloquent brain (indirect feeder), in which case MEP/SSEP deterioration will occur within minutes [5, 9, 13]. This guided us in several cases to safely sacrifice small feeding branches while preserving larger arteries that turned out to supply functional cortex. Such fine distinctions, often impossible to make by visual inspection alone, reduce the risk of inadvertent ischemic stroke in remote areas [5, 9, 13]. This principle is analogous to IONM use in aneurysm surgery, where monitoring during temporary clipping of parent vessels or perforators can detect ischemia early. By incorporating similar techniques in AVM resection, we can confidently pursue nidus devascularization while avoiding occlusion of “innocent” bystander arteries, thereby minimizing collateral damage [5, 9, 13].

Depending on the AVM’s location, additional monitoring modalities are employed to safeguard neurological function. For AVMs near the brainstem or internal auditory canal, brainstem auditory evoked potentials (BAEPs) are monitored to warn of brainstem or auditory nerve stress. In occipital lobe or temporo-occipital AVMs, visual evoked potentials (VEPs) can be used to monitor optic pathway integrity [37]. Cranial nerve function is observed via triggered or free-running electromyography (EMG) for AVMs in the vicinity of the cranial nerves. While these specialized modalities are not needed in every case, they provide targeted protection for functions that are not covered by MEPs/SSEPs. For instance, in the study by Zhou et al., BAEP monitoring was found to complement SSEPs and MEPs, particularly in grade III–IV AVMs, by detecting brainstem stress that motor/sensory potentials might not catch [34, 37]. Overall, the multimodal IONM approach can be tailored to each AVM’s anatomic context, ensuring that whether the risk is to movement, sensation, language, vision, or hearing, the surgical team has a real-time indicator of neurological well-being.

Crucially, the use of these mapping and monitoring techniques means that the surgeon is never *“operating in the dark”* with respect to the patient’s functional status [13, 33]. The net result is that we can confidently tackle AVMs in eloquent or deep locations that might have been deemed inoperable in the past, with a high expectation of preserving neurological function. Our data demonstrates that with IONM guidance, the outcomes for high-risk AVMs have effectively caught up to those of low-risk AVMs, validating the expanded role of surgery in these challenging cases.

### Comparison with our previous institutional experience

Although a direct comparison with non-monitored cohorts could theoretically strengthen causal inference, such an approach was not pursued as a primary objective of the present study. Historical institutional data were only partially retrievable and characterized by substantial heterogeneity in surgical techniques, perioperative management, and operator experience. In contrast, the current series represents a homogeneous cohort of AVMs treated by a single surgical team, allowing for consistent application of both surgical strategy and intraoperative neurophysiological monitoring protocols.

To provide contextual insight, we retrospectively reviewed cases from the five-year period preceding the study that would have been classified as high motor-risk according to the proposed framework. Among these cases (*n* = 19), postoperative motor deficits were observed in four patients. Of these, one AVM was classified as high topographic risk due to involvement of the pre-Rolandic region, whereas the remaining three cases were primarily characterized by vascular risk, being supplied by en passant perforating branches arising from the proximal middle cerebral artery. These findings support the central premise of the study, namely that functional risk is not solely determined by AVM size or Spetzler–Martin grade, but also by procedure-specific vascular anatomy and the presence of critical bystander vessels.

Overall, two permanent motor deficits and two transient motor deficits were observed, the latter resolving completely by six-month follow-up. Additionally, two small, unanticipated residual AVMs were identified and subsequently treated with radiosurgery, resulting in progressive thrombosis. These observations are reported for descriptive purposes only but cannot be interpreted as evidence of comparative efficacy.

Notably, before the routine implementation of intraoperative neurophysiological monitoring, several AVMs considered at high topographic risk, particularly those adjacent to the Rolandic cortex, were preferentially referred for radiosurgical treatment. The systematic use of monitoring has progressively allowed a shift in surgical decision-making, enabling complete and definitive microsurgical resection in selected high-risk cases while maintaining an acceptable functional safety profile. Although this observation remains descriptive, it suggests that IONM may contribute to expanding surgical indications in anatomically complex AVMs by mitigating motor risk.

The present study suggests that the integration of intraoperative neurophysiological monitoring into a risk-oriented surgical strategy may influence the functional risk profile traditionally associated with eloquent AVMs. In our series, AVMs classified as high motor-risk based on anatomical and vascular criteria achieved postoperative functional outcomes comparable to those observed in low-risk, non-eloquent lesions. This finding does not imply that eloquent AVMs are intrinsically low risk, but rather that real-time functional feedback provided by IONM can inform intraoperative decision-making, allowing the surgeon to modulate the extent of resection according to procedure-specific vulnerability. In this context, IONM acts as a dynamic tool to identify critical motor pathways and bystander vessels, facilitating a “maximal safe resection” strategy focused on functional preservation rather than angiographic radicality alone.

### Limitations

Despite its clear benefits, IONM is not a panacea, and both technical and practical limitations must be acknowledged. False-positive alerts can occur due to anesthetic fluctuations, systemic factors, or technical issues. For instance, SSEPs can fluctuate with changes in anesthetic depth, blood pressure, or body temperature. MEP signals are even more finicky: they are highly sensitive to inhalational anesthetics, neuromuscular blockade, and electrode montage [38]. Subtle shifts in electrode contact or in serum electrolyte levels can increase MEP thresholds or cause loss of response. Therefore, interpretation of IONM changes requires expertise. Overinterpreting minor, transient changes could lead to unnecessary pauses or premature abortion of resection [38]. In our series, close communication with the neurophysiologist was essential; on a few occasions, SSEP or MEP changes were noted, but after troubleshooting, adjusting anesthesia, checking leads, or increasing blood pressure, the signals recovered, and surgery continued uneventfully. Such instances highlight the importance of maintaining optimal monitoring conditions to maximize the reliability of IONM [38].

Another consideration is that IONM may not improve outcomes for every AVM patient, particularly those with low-risk lesions. Zhou et al. found that, overall, complication rates were not statistically different between monitored and unmonitored groups. The clearest benefits of IONM were seen in higher-grade (SM grade ≥ III) AVMs, whereas small superficial AVMs (grades I–II) had good outcomes even without monitoring [5, 9, 13]. This suggests that routine IONM for every AVM case might not be cost-effective or necessary; patient selection is key. In practice, we now tailor the intensity of monitoring to the case complexity, for a small lobar AVM far from eloquent cortex, a basic setup (e.g. SSEPs alone) may suffice, whereas a large diffuse AVM in the peri-Rolandic region warrants full multimodality monitoring and mapping [5, 9, 13, 36]. Surgeons must also be prepared for the scenario where, despite all precautions, an intraoperative rupture or sudden hemodynamic change forces a swift completion of resection. In such emergencies, monitoring alerts may come too late or be overshadowed by the need to control hemorrhage. Fortunately, catastrophic bleeds were not encountered in our series, but the risk exists, and it underscores that IONM is an aid, not a replacement for surgical judgment and skill. Finally, it’s worth noting that leaving a residual nidus, while sometimes necessary, does carry a risk of re-bleeding until the AVM is definitively obliterated. We mitigated this by coupling this with a clear plan for definitive treatment of the residual and close monitoring in the interim. Patients and families must be advised about this trade-off: a slightly higher short-term hemorrhage risk versus the benefit of avoiding a permanent deficit. In our view, for carefully selected cases, this trade-off is justified, preserving neurological function is paramount, and modern adjuncts like radiosurgery can effectively manage most residual AVMs.

Finally, it is important to consider that it is a single-center experience with a relatively small sample size that may limit the generalizability of these findings. Moreover, the absence of a contemporaneous control group of high-risk patients treated without IONM precludes definitive causal attribution of the observed safety profile to monitoring alone. Historical comparisons were not designed as a formal analytical component of the study due to incomplete data availability and heterogeneity in surgical techniques and operator experience across different time periods.

## Conclusions

In our institutional experience, IONM plays a pivotal role in the safe resection of AVMs located in high motor-risk areas. By providing real-time functional feedback, IONM enables us to accurately identify and respect functional boundaries, thereby minimizing the risk of iatrogenic neurological injury, thus downgrading a high motor risk in a low motor risk AVMs located in eloquent areas. IONM may support an intraoperative decision to pursue a subtotal resection, to be eventually followed by adjuvant radiosurgery, allowing to refine a tailored strategy prioritizing long-term functional preservation in selected cases. In particular, our findings support a risk-adapted IONM strategy, in which the intensity and modality of monitoring are tailored to the anatomical and functional risk profile of the AVM, without compromising patient safety.

## Data Availability

Available upon reasonable request.
